# Phenyl 2-amino-*N*,6-*O*-dibenzyl-2,3-*N*,*O*-carbonyl-2-de­oxy-1-thio-β-d-glucopyran­oside

**DOI:** 10.1107/S1600536808027189

**Published:** 2008-09-06

**Authors:** Shino Manabe, Kazuyuki Ishii, Daisuke Hashizume, Yukishige Ito

**Affiliations:** a Advanced Science Institute, RIKEN (The Institute of Physical and Chemical Research), Hirosawa, Wako, Saitama 351-0198, Japan; b Advanced Technology Support Division, RIKEN (The Institute of Physical and Chemical Research), Hirosawa, Wako, Saitama 351-0198, Japan

## Abstract

In the crystal structure of the title compound, C_27_H_27_NO_5_S, the pyran­ose ring adopts a ^4^ *C*_1_ chair conformation with puckering parameters *Q* = 0.639 (2) Å, *θ* = 174.11 (18) and ϕ = 256 (2)°. The presence of the 2,3-*trans-*oxazolidinone fixes the conformation of the pyran­ose ring. The phenyl group attached to the S atom and the benzyl group bonding to the N atom are each disordered over two positions with site occupancies of 0.624 (3):0.376 (3) and 0.526 (3):0.474 (3), respectively. An inter­molecular O—H⋯O hydrogen bond is observed.

## Related literature

For related literature, see: Benakli *et al.* (2001 ▶); Boysen *et al.* (2005 ▶); Cremer & Pople (1975 ▶); Crich & Vinod (2005 ▶); Geng *et al.* (2008 ▶); Manabe *et al.* (2006 ▶); Satoh *et al.* (2008 ▶).
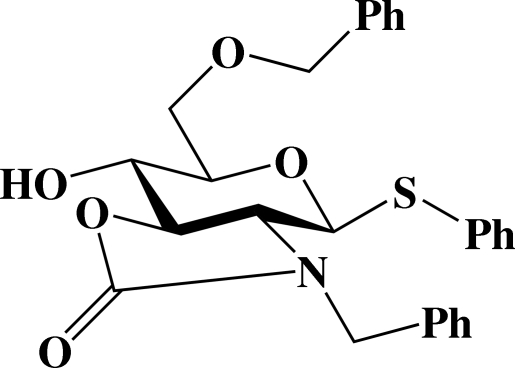


## Experimental

### 

#### Crystal data

C_27_H_27_NO_5_S
*M* *_r_* = 477.56
Monoclinic, 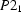

*a* = 13.8166 (7) Å
*b* = 5.7008 (3) Å
*c* = 15.0425 (9) Å
β = 91.494 (4)°
*V* = 1184.43 (11) Å^3^
*Z* = 2
Mo *K*α radiation
μ = 0.18 mm^−1^
*T* = 90 K
0.30 × 0.08 × 0.07 mm

#### Data collection

Rigaku AFC8 diffractometer with Saturn70 CCD
Absorption correction: multi-scan (**MULABS**; Blessing, 1995 ▶) *T*_min_ = 0.959, *T*_max_ = 0.998
7074 measured reflections
4965 independent reflections
3970 reflections with *I* > 2σ(*I*)
*R*_int_ = 0.040

#### Refinement

*R*[*F* ^2^ > 2σ(*F* ^2^)] = 0.058
*wR*(*F* ^2^) = 0.167
*S* = 1.03
4965 reflections
353 parameters
31 restraints
H-atom parameters constrained
Δρ_max_ = 0.33 e Å^−3^
Δρ_min_ = −0.34 e Å^−3^
Absolute structure: Flack (1983 ▶), 1983 Friedel pairs
Flack parameter: 0.05 (11)

### 

Data collection: *CrystalClear SM* (Rigaku/MSC, 2005 ▶); cell refinement: *HKL-2000* (Otwinowski & Minor, 1997 ▶); data reduction: *HKL-2000*; program(s) used to solve structure: *SIR2004* (Burla *et al.*, 2005 ▶); program(s) used to refine structure: *SHELXL97* (Sheldrick, 2008 ▶); molecular graphics: *ORTEP-3 for Windows* (Farrugia, 1997 ▶); software used to prepare material for publication: *SHELXL97*.

## Supplementary Material

Crystal structure: contains datablocks I, global. DOI: 10.1107/S1600536808027189/is2324sup1.cif

Structure factors: contains datablocks I. DOI: 10.1107/S1600536808027189/is2324Isup2.hkl

Additional supplementary materials:  crystallographic information; 3D view; checkCIF report

## Figures and Tables

**Table 1:** Hydrogen-bond geometry (Å, °)

*D*—H⋯*A*	*D*—H	H⋯*A*	*D*⋯*A*	*D*—H⋯*A*
O3—H3*O*⋯O5^i^	0.84	1.98	2.774 (2)	158

Symmetry code: (i) 
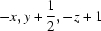
.

**Fig. 1.**
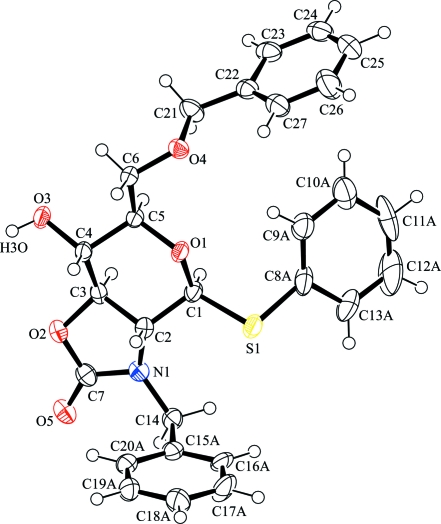
A view of the molecular structure of (I), showing the atom labeling scheme. Displacement ellipsoids are drawn at the 50% probability level. The minor parts of the disordered phenyl moieties are omitted for clarity.

## supplementary crystallographic information

### Comment

Recently, we and other groups reported 2,3-*trans*-oxazolidinone carrying novel glycosyl donors for α-selective glycosylation (Benakli *et al.*, 2001; Boysen *et al.*, 2005; Crich & Vinod, 2005; Manabe *et al.*, 2006; Geng *et al.*, 2008). Furthermore, we also found the 2,3-*trans*-oxazolidinone carrying pyranoses are easily anomerized *via* an *endo*-cleavage manner (Manabe *et al.*, 2006; Satoh *et al.*, 2008). In order to investigate the origin of the high stereosetectivity and its unique character, we are interested in the conformation of the pyranose ring with 2,3-*trans-*oxazolidinone group.

The pyranose ring of the title compound adapts a ^4^*C*_1_ chair conformation. The torsion angles (O1—C1—C2—C3, C1—C2—C3—C4, C2—C3—C4—C5, C3—C4—C5—O1, C4—C5—O1—C1 and C5—O1—C1—C2; see geometric parameters table in supplementary materials) and Cremer-Pople puckering parameters (Cremer & Pople, 1975), *Q* = 0.639 (2) Å, *θ* = 174.11 (18)° and φ = 256 (2)°, of the ring clearly indicate the ^4^*C*_1_ conformation.

### Experimental

The compound, (I), was prepared as described by Benakli *et al.* (2001). (I) was dissolved in ethyl acetate at room temperature and hexane was added. The solution was kept at room temperature in a sealed flask for a few days to give single crystals suitable for single-crystal X-ray analysis.

### Refinement

The phenyl group bonding to the S atom and the benzyl group bonding to the N atom in (I) were disordered into two parts each other. Occupancy factors of each group were refined, where the atomic displacement parameters of the corresponding atoms in each group were constrained to be the same. Restraints were imposed on the C—C bond distances in the disordered phenyl groups to be 1.39 Å. The positions of H atoms on C atoms were calculated from geometries. The H atom of the hydroxyl group was located in a difference map. All H atoms were treated as riding atoms with C/O—H distances of 1.00, 0.99, 0.95 and 0.84 Å for methyne, methylene, phenyl and hydroxyl, respectively. The *U*_iso_(H) values were fixed to be 1.2*U*_eq_(C) or 1.5*U*_eq_(O).

### Crystal data

**Table:**

C_27_H_27_NO_5_S	*F*(000) = 504
*M**_r_* = 477.56	*D*_x_ = 1.339 Mg m^−^^3^
Monoclinic, *P*2_1_	Mo *K*α radiation, λ = 0.71073 Å
Hall symbol: P 2yb	Cell parameters from 7082 reflections
*a* = 13.8166 (7) Å	θ = 1.4–27.5°
*b* = 5.7008 (3) Å	µ = 0.18 mm^−^^1^
*c* = 15.0425 (9) Å	*T* = 90 K
β = 91.494 (4)°	Needle, colourless
*V* = 1184.43 (11) Å^3^	0.30 × 0.08 × 0.07 mm
*Z* = 2	

### Data collection

**Table:**

Rigaku AFC8 diffractometer with Saturn70 CCD	4965 independent reflections
Radiation source: fine-focus rotating anode	3970 reflections with *I* > 2σ(*I*)
confocal	*R*_int_ = 0.040
Detector resolution: 28.5714 pixels mm^-1^	θ_max_ = 27.5°, θ_min_ = 1.4°
ω–scan	*h* = −17→15
Absorption correction: multi-scan (*MULABS*; Blessing, 1995)	*k* = −7→6
*T*_min_ = 0.959, *T*_max_ = 0.998	*l* = −19→19
7074 measured reflections	

### Refinement

**Table:**

Refinement on *F*^2^	Secondary atom site location: difference Fourier map
Least-squares matrix: full	Hydrogen site location: inferred from neighbouring sites
*R*[*F*^2^ > 2σ(*F*^2^)] = 0.058	H-atom parameters constrained
*wR*(*F*^2^) = 0.167	*w* = 1/[σ^2^(*F*_o_^2^) + (0.0942*P*)^2^] where *P* = (*F*_o_^2^ + 2*F*_c_^2^)/3
*S* = 1.03	(Δ/σ)_max_ = 0.011
4965 reflections	Δρ_max_ = 0.33 e Å^−^^3^
353 parameters	Δρ_min_ = −0.34 e Å^−^^3^
31 restraints	Absolute structure: Flack (1983), 1983 Friedel pairs
Primary atom site location: structure-invariant direct methods	Flack parameter: 0.05 (11)

### Special details

**Table:**

Geometry. All e.s.d.'s (except the e.s.d. in the dihedral angle between two l.s. planes) are estimated using the full covariance matrix. The cell e.s.d.'s are taken into account individually in the estimation of e.s.d.'s in distances, angles and torsion angles; correlations between e.s.d.'s in cell parameters are only used when they are defined by crystal symmetry. An approximate (isotropic) treatment of cell e.s.d.'s is used for estimating e.s.d.'s involving l.s. planes.
Refinement. Refinement of *F*^2^ against all reflections. The weighted *R*-factor *wR* and goodness of fit *S* are based on *F*^2^, conventional *R*-factors *R* are based on *F*, with *F* set to zero for negative *F*^2^. The threshold expression of *F*^2^ > 2σ(*F*^2^) is used only for calculating *R*-factors(gt) *etc*. and is not relevant to the choice of reflections for refinement. *R*-factors based on *F*^2^ are statistically about twice as large as those based on *F*, and *R*- factors based on all data will be even larger.

### Fractional atomic coordinates and isotropic or equivalent isotropic displacement parameters (Å^2^)

**Table:**

	*x*	*y*	*z*	*U*_iso_*/*U*_eq_	Occ. (<1)
S1	0.34405 (4)	0.61596 (13)	0.35271 (4)	0.03602 (14)	
O1	0.16119 (10)	0.6528 (3)	0.30101 (9)	0.0265 (3)	
O2	0.07008 (11)	0.0779 (3)	0.44133 (9)	0.0280 (4)	
O3	−0.06858 (11)	0.3322 (3)	0.31331 (10)	0.0294 (4)	
H3O	−0.1054	0.3031	0.3555	0.035*	
O4	0.05569 (11)	0.9160 (3)	0.17927 (9)	0.0272 (4)	
O5	0.16411 (12)	−0.1618 (3)	0.52679 (10)	0.0333 (4)	
N1	0.22987 (13)	0.1425 (3)	0.44686 (11)	0.0266 (4)	
C1	0.23048 (16)	0.4742 (4)	0.32615 (15)	0.0268 (5)	
H1	0.2383	0.3602	0.2763	0.032*	
C2	0.18671 (15)	0.3529 (4)	0.40565 (13)	0.0245 (5)	
H2	0.1728	0.4714	0.4526	0.029*	
C3	0.09332 (16)	0.2431 (4)	0.37230 (13)	0.0254 (5)	
H3	0.1076	0.1517	0.3174	0.030*	
C4	0.01933 (15)	0.4275 (4)	0.34745 (13)	0.0247 (5)	
H4	0.0072	0.5329	0.3991	0.030*	
C5	0.06745 (15)	0.5641 (4)	0.27204 (13)	0.0240 (5)	
H5	0.0760	0.4565	0.2203	0.029*	
C6	0.00733 (16)	0.7723 (4)	0.24210 (14)	0.0259 (5)	
H61	−0.0544	0.7155	0.2149	0.031*	
H62	−0.0084	0.8683	0.2946	0.031*	
C7	0.15701 (17)	0.0048 (4)	0.47591 (14)	0.0293 (5)	
C8A	0.39021 (14)	0.6625 (6)	0.24481 (13)	0.0433 (7)	0.624 (3)
C9A	0.3372 (2)	0.7700 (7)	0.17667 (14)	0.0450 (10)	0.624 (3)
H9A	0.2725	0.8185	0.1862	0.054*	0.624 (3)
C10A	0.37834 (16)	0.8070 (8)	0.09447 (18)	0.0571 (11)	0.624 (3)
H10A	0.3428	0.8712	0.0453	0.068*	0.624 (3)
C11A	0.47501 (18)	0.7437 (8)	0.0892 (2)	0.0800 (12)	0.624 (3)
H11A	0.5053	0.7669	0.0339	0.096*	0.624 (3)
C12A	0.5310 (2)	0.6490 (10)	0.15830 (17)	0.094 (2)	0.624 (3)
H12A	0.5972	0.6115	0.1502	0.113*	0.624 (3)
C13A	0.48825 (16)	0.6104 (10)	0.2395 (2)	0.0628 (13)	0.624 (3)
H13A	0.5243	0.5510	0.2892	0.075*	0.624 (3)
C8B	0.3898 (2)	0.6505 (5)	0.24391 (14)	0.0433 (7)	0.376 (3)
C9B	0.3554 (3)	0.8270 (7)	0.18792 (19)	0.0450 (10)	0.376 (3)
H9B	0.3031	0.9236	0.2052	0.054*	0.376 (3)
C10B	0.3982 (3)	0.8608 (9)	0.1063 (2)	0.0571 (11)	0.376 (3)
H10B	0.3778	0.9961	0.0740	0.068*	0.376 (3)
C11B	0.4669 (4)	0.7218 (6)	0.0659 (3)	0.0800 (12)	0.376 (3)
H11B	0.4979	0.7616	0.0124	0.096*	0.376 (3)
C12B	0.4845 (4)	0.5173 (8)	0.1138 (2)	0.094 (2)	0.376 (3)
H12B	0.5231	0.3964	0.0896	0.113*	0.376 (3)
C13B	0.4453 (3)	0.4903 (8)	0.1974 (2)	0.0628 (13)	0.376 (3)
H13B	0.4580	0.3449	0.2263	0.075*	0.376 (3)
C14	0.323625 (11)	0.13833 (2)	0.49395 (2)	0.0290 (5)	
H141	0.3748	0.1717	0.4509	0.035*	
H142	0.3350	−0.0217	0.5175	0.035*	
C15A	0.333196 (11)	0.31100 (2)	0.56988 (2)	0.0311 (5)	0.526 (3)
C16A	0.410308 (11)	0.46761 (2)	0.56994 (2)	0.0464 (11)	0.526 (3)
H16A	0.4574	0.4574	0.5252	0.056*	0.526 (3)
C17A	0.4187 (2)	0.6396 (6)	0.63559 (18)	0.0654 (15)	0.526 (3)
H17A	0.4742	0.7381	0.6368	0.078*	0.526 (3)
C18A	0.34885 (18)	0.6723 (6)	0.6995 (2)	0.0455 (12)	0.526 (3)
H18A	0.3538	0.7955	0.7419	0.055*	0.526 (3)
C19A	0.2716 (2)	0.5160 (5)	0.6983 (2)	0.0371 (7)	0.526 (3)
H19A	0.2237	0.5283	0.7422	0.045*	0.526 (3)
C20A	0.2633 (2)	0.3421 (6)	0.63375 (17)	0.0306 (5)	0.526 (3)
H20A	0.2084	0.2417	0.6332	0.037*	0.526 (3)
C15B	0.33272 (2)	0.30995 (3)	0.57017 (3)	0.0311 (5)	0.474 (3)
C16B	0.42307 (17)	0.4031 (7)	0.5928 (2)	0.0464 (11)	0.474 (3)
H16B	0.4764	0.3710	0.5561	0.056*	0.474 (3)
C17B	0.4369 (2)	0.5420 (9)	0.6679 (2)	0.0654 (15)	0.474 (3)
H17B	0.4978	0.6131	0.6812	0.078*	0.474 (3)
C18B	0.35861 (16)	0.5736 (9)	0.7230 (3)	0.0455 (12)	0.474 (3)
H18B	0.3673	0.6613	0.7763	0.055*	0.474 (3)
C19B	0.2680 (2)	0.4799 (6)	0.7020 (2)	0.0371 (7)	0.474 (3)
H19B	0.2147	0.5114	0.7388	0.045*	0.474 (3)
C20B	0.25570 (19)	0.3397 (8)	0.62686 (19)	0.0306 (5)	0.474 (3)
H20B	0.1954	0.2652	0.6144	0.037*	0.474 (3)
C21	0.0574 (2)	0.8149 (4)	0.09276 (14)	0.0355 (6)	
H211	−0.0091	0.8092	0.0667	0.043*	
H212	0.0824	0.6525	0.0967	0.043*	
C22	0.12116 (17)	0.9594 (4)	0.03426 (14)	0.0297 (5)	
C23	0.13491 (18)	0.8889 (5)	−0.05315 (14)	0.0359 (6)	
H23	0.1038	0.7511	−0.0749	0.043*	
C24	0.1933 (2)	1.0167 (5)	−0.10881 (16)	0.0434 (7)	
H24	0.2033	0.9649	−0.1679	0.052*	
C25	0.2369 (2)	1.2195 (6)	−0.07797 (18)	0.0572 (9)	
H25	0.2758	1.3094	−0.1164	0.069*	
C26	0.2243 (2)	1.2937 (6)	0.0092 (2)	0.0610 (9)	
H26	0.2543	1.4335	0.0304	0.073*	
C27	0.1669 (2)	1.1598 (5)	0.06496 (15)	0.0405 (7)	
H27	0.1592	1.2074	0.1249	0.049*	

### Atomic displacement parameters (Å^2^)

**Table:**

	*U*^11^	*U*^22^	*U*^33^	*U*^12^	*U*^13^	*U*^23^
S1	0.0211 (2)	0.0387 (3)	0.0481 (3)	−0.0025 (3)	−0.0012 (2)	0.0103 (3)
O1	0.0172 (6)	0.0286 (7)	0.0336 (7)	−0.0016 (6)	0.0000 (6)	0.0039 (6)
O2	0.0292 (7)	0.0257 (8)	0.0292 (7)	−0.0036 (6)	0.0067 (6)	0.0029 (6)
O3	0.0201 (7)	0.0382 (8)	0.0299 (7)	−0.0081 (7)	0.0018 (6)	−0.0026 (7)
O4	0.0296 (8)	0.0303 (7)	0.0216 (6)	−0.0015 (7)	0.0019 (6)	−0.0008 (6)
O5	0.0404 (9)	0.0290 (8)	0.0311 (7)	0.0043 (7)	0.0105 (7)	0.0035 (6)
N1	0.0280 (9)	0.0252 (8)	0.0268 (8)	0.0022 (8)	0.0026 (7)	0.0008 (7)
C1	0.0191 (10)	0.0263 (10)	0.0350 (11)	0.0010 (9)	0.0003 (9)	0.0046 (9)
C2	0.0233 (10)	0.0238 (10)	0.0264 (9)	−0.0009 (9)	0.0026 (8)	−0.0017 (8)
C3	0.0256 (10)	0.0274 (10)	0.0235 (9)	−0.0059 (9)	0.0064 (8)	−0.0023 (8)
C4	0.0214 (10)	0.0303 (10)	0.0224 (9)	−0.0066 (9)	0.0024 (8)	−0.0031 (8)
C5	0.0201 (9)	0.0282 (11)	0.0238 (9)	−0.0028 (8)	0.0004 (8)	−0.0008 (8)
C6	0.0203 (10)	0.0303 (11)	0.0270 (10)	−0.0034 (9)	0.0003 (9)	0.0005 (8)
C7	0.0348 (12)	0.0273 (10)	0.0263 (10)	0.0019 (10)	0.0079 (9)	−0.0019 (8)
C8A	0.0235 (11)	0.0399 (13)	0.0672 (15)	0.0004 (11)	0.0129 (11)	0.0111 (12)
C9A	0.0326 (17)	0.060 (2)	0.0426 (15)	−0.0028 (18)	0.0067 (14)	−0.0048 (16)
C10A	0.059 (2)	0.069 (3)	0.0444 (16)	−0.024 (2)	0.0153 (17)	−0.0122 (18)
C11A	0.085 (2)	0.078 (2)	0.080 (2)	−0.016 (2)	0.0654 (18)	−0.004 (2)
C12A	0.044 (2)	0.116 (5)	0.125 (4)	0.012 (3)	0.049 (2)	0.001 (4)
C13A	0.0211 (17)	0.092 (3)	0.076 (3)	0.001 (2)	0.0054 (17)	0.002 (3)
C8B	0.0235 (11)	0.0399 (13)	0.0672 (15)	0.0004 (11)	0.0129 (11)	0.0111 (12)
C9B	0.0326 (17)	0.060 (2)	0.0426 (15)	−0.0028 (18)	0.0067 (14)	−0.0048 (16)
C10B	0.059 (2)	0.069 (3)	0.0444 (16)	−0.024 (2)	0.0153 (17)	−0.0122 (18)
C11B	0.085 (2)	0.078 (2)	0.080 (2)	−0.016 (2)	0.0654 (18)	−0.004 (2)
C12B	0.044 (2)	0.116 (5)	0.125 (4)	0.012 (3)	0.049 (2)	0.001 (4)
C13B	0.0211 (17)	0.092 (3)	0.076 (3)	0.001 (2)	0.0054 (17)	0.002 (3)
C14	0.0238 (9)	0.0316 (11)	0.0317 (10)	0.0071 (10)	0.0045 (8)	0.0026 (10)
C15A	0.0252 (11)	0.0408 (12)	0.0271 (10)	0.0066 (10)	−0.0008 (9)	0.0045 (9)
C16A	0.0222 (14)	0.095 (3)	0.0218 (16)	−0.0046 (18)	−0.0064 (13)	−0.003 (2)
C17A	0.0294 (17)	0.134 (4)	0.033 (2)	−0.021 (2)	−0.0030 (17)	−0.025 (2)
C18A	0.0459 (18)	0.061 (3)	0.0296 (17)	−0.009 (2)	0.0044 (15)	−0.0054 (18)
C19A	0.0285 (12)	0.0524 (17)	0.0306 (11)	−0.0031 (12)	0.0037 (10)	−0.0021 (11)
C20A	0.0249 (11)	0.0373 (12)	0.0296 (11)	0.0044 (10)	−0.0006 (10)	0.0028 (10)
C15B	0.0252 (11)	0.0408 (12)	0.0271 (10)	0.0066 (10)	−0.0008 (9)	0.0045 (9)
C16B	0.0222 (14)	0.095 (3)	0.0218 (16)	−0.0046 (18)	−0.0064 (13)	−0.003 (2)
C17B	0.0294 (17)	0.134 (4)	0.033 (2)	−0.021 (2)	−0.0030 (17)	−0.025 (2)
C18B	0.0459 (18)	0.061 (3)	0.0296 (17)	−0.009 (2)	0.0044 (15)	−0.0054 (18)
C19B	0.0285 (12)	0.0524 (17)	0.0306 (11)	−0.0031 (12)	0.0037 (10)	−0.0021 (11)
C20B	0.0249 (11)	0.0373 (12)	0.0296 (11)	0.0044 (10)	−0.0006 (10)	0.0028 (10)
C21	0.0492 (14)	0.0344 (11)	0.0231 (10)	−0.0067 (11)	0.0037 (10)	−0.0035 (9)
C22	0.0289 (11)	0.0328 (11)	0.0275 (10)	0.0072 (10)	0.0006 (9)	0.0023 (9)
C23	0.0359 (12)	0.0443 (13)	0.0275 (10)	0.0135 (11)	0.0000 (10)	0.0008 (10)
C24	0.0365 (13)	0.0675 (16)	0.0263 (11)	0.0197 (13)	0.0039 (10)	0.0069 (11)
C25	0.0511 (17)	0.087 (2)	0.0340 (13)	−0.0145 (17)	0.0034 (13)	0.0172 (14)
C26	0.068 (2)	0.0681 (19)	0.0474 (15)	−0.0265 (17)	0.0064 (15)	0.0103 (14)
C27	0.0449 (14)	0.0462 (14)	0.0305 (11)	−0.0062 (12)	0.0009 (11)	0.0041 (11)

### Geometric parameters (Å, °)

**Table:**

S1—C8A	1.7791 (19)	C11B—H11B	0.9500
S1—C8B	1.781 (2)	C12B—C13B	1.390 (3)
S1—C1	1.801 (2)	C12B—H12B	0.9500
O1—C1	1.441 (3)	C13B—H13B	0.9500
O1—C5	1.447 (2)	C14—C15B	1.5099 (4)
O2—C7	1.362 (3)	C14—C15A	1.5110 (4)
O2—C3	1.444 (2)	C14—H141	0.9900
O3—C4	1.415 (3)	C14—H142	0.9900
O3—H3O	0.8400	C15A—C16A	1.3900 (2)
O4—C21	1.424 (3)	C15A—C20A	1.391 (2)
O4—C6	1.430 (3)	C16A—C17A	1.394 (2)
O5—C7	1.222 (3)	C16A—H16A	0.9500
N1—C7	1.358 (3)	C17A—C18A	1.393 (3)
N1—C14	1.4604 (18)	C17A—H17A	0.9500
N1—C2	1.470 (3)	C18A—C19A	1.390 (3)
C1—C2	1.520 (3)	C18A—H18A	0.9500
C1—H1	1.0000	C19A—C20A	1.390 (3)
C2—C3	1.508 (3)	C19A—H19A	0.9500
C2—H2	1.0000	C20A—H20A	0.9500
C3—C4	1.507 (3)	C15B—C16B	1.391 (2)
C3—H3	1.0000	C15B—C20B	1.391 (2)
C4—C5	1.541 (3)	C16B—C17B	1.389 (3)
C4—H4	1.0000	C16B—H16B	0.9500
C5—C6	1.511 (3)	C17B—C18B	1.391 (3)
C5—H5	1.0000	C17B—H17B	0.9500
C6—H61	0.9900	C18B—C19B	1.390 (3)
C6—H62	0.9900	C18B—H18B	0.9500
C8A—C9A	1.387 (2)	C19B—C20B	1.390 (3)
C8A—C13A	1.391 (2)	C19B—H19B	0.9500
C9A—C10A	1.390 (2)	C20B—H20B	0.9500
C9A—H9A	0.9500	C21—C22	1.507 (3)
C10A—C11A	1.388 (2)	C21—H211	0.9900
C10A—H10A	0.9500	C21—H212	0.9900
C11A—C12A	1.388 (3)	C22—C27	1.379 (3)
C11A—H11A	0.9500	C22—C23	1.393 (3)
C12A—C13A	1.388 (3)	C23—C24	1.385 (4)
C12A—H12A	0.9500	C23—H23	0.9500
C13A—H13A	0.9500	C24—C25	1.379 (4)
C8B—C9B	1.388 (3)	C24—H24	0.9500
C8B—C13B	1.393 (3)	C25—C26	1.393 (4)
C9B—C10B	1.390 (3)	C25—H25	0.9500
C9B—H9B	0.9500	C26—C27	1.397 (4)
C10B—C11B	1.389 (3)	C26—H26	0.9500
C10B—H10B	0.9500	C27—H27	0.9500
C11B—C12B	1.389 (3)		
			
C8A—S1—C1	101.28 (11)	C9B—C10B—H10B	115.9
C8B—S1—C1	100.03 (13)	C10B—C11B—C12B	111.4 (4)
C1—O1—C5	114.53 (15)	C10B—C11B—H11B	124.3
C7—O2—C3	105.30 (16)	C12B—C11B—H11B	124.3
C4—O3—H3O	109.5	C11B—C12B—C13B	119.7 (4)
C21—O4—C6	113.05 (17)	C11B—C12B—H12B	120.2
C7—N1—C14	119.35 (15)	C13B—C12B—H12B	120.2
C7—N1—C2	108.16 (17)	C12B—C13B—C8B	127.9 (4)
C14—N1—C2	124.26 (15)	C12B—C13B—H13B	116.1
O1—C1—C2	104.63 (16)	C8B—C13B—H13B	116.1
O1—C1—S1	108.08 (14)	N1—C14—C15B	114.39 (7)
C2—C1—S1	113.07 (15)	N1—C14—C15A	114.53 (7)
O1—C1—H1	110.3	N1—C14—H141	108.6
C2—C1—H1	110.3	N1—C14—H142	108.6
S1—C1—H1	110.3	C16A—C15A—C20A	117.64 (13)
N1—C2—C3	97.80 (16)	C16A—C15A—C14	118.1
N1—C2—C1	122.53 (17)	C20A—C15A—C14	123.79 (12)
C3—C2—C1	106.35 (17)	C15A—C16A—C17A	120.10 (12)
N1—C2—H2	109.7	C15A—C16A—H16A	119.9
C3—C2—H2	109.7	C17A—C16A—H16A	119.9
C1—C2—H2	109.7	C18A—C17A—C16A	122.5 (3)
O2—C3—C4	118.12 (17)	C18A—C17A—H17A	118.8
O2—C3—C2	103.64 (16)	C16A—C17A—H17A	118.8
C4—C3—C2	111.22 (17)	C19A—C18A—C17A	116.8 (3)
O2—C3—H3	107.8	C19A—C18A—H18A	121.6
C4—C3—H3	107.8	C17A—C18A—H18A	121.6
C2—C3—H3	107.8	C18A—C19A—C20A	121.1 (3)
O3—C4—C3	113.12 (17)	C18A—C19A—H19A	119.5
O3—C4—C5	108.03 (16)	C20A—C19A—H19A	119.5
C3—C4—C5	103.43 (16)	C19A—C20A—C15A	121.8 (3)
O3—C4—H4	110.7	C19A—C20A—H20A	119.1
C3—C4—H4	110.7	C15A—C20A—H20A	119.1
C5—C4—H4	110.7	C16B—C15B—C20B	119.96 (18)
O1—C5—C6	107.21 (16)	C16B—C15B—C14	119.31 (13)
O1—C5—C4	110.79 (16)	C20B—C15B—C14	119.65 (14)
C6—C5—C4	111.84 (17)	C17B—C16B—C15B	121.4 (2)
O1—C5—H5	109.0	C17B—C16B—H16B	119.3
C6—C5—H5	109.0	C15B—C16B—H16B	119.3
C4—C5—H5	109.0	C16B—C17B—C18B	117.7 (3)
O4—C6—C5	112.61 (17)	C16B—C17B—H17B	121.1
O4—C6—H61	109.1	C18B—C17B—H17B	121.1
C5—C6—H61	109.1	C19B—C18B—C17B	121.6 (3)
O4—C6—H62	109.1	C19B—C18B—H18B	119.2
C5—C6—H62	109.1	C17B—C18B—H18B	119.2
H61—C6—H62	107.8	C18B—C19B—C20B	119.7 (3)
O5—C7—N1	127.1 (2)	C18B—C19B—H19B	120.1
O5—C7—O2	122.2 (2)	C20B—C19B—H19B	120.1
N1—C7—O2	110.73 (18)	C19B—C20B—C15B	119.3 (3)
C9A—C8A—C13A	123.2 (2)	C19B—C20B—H20B	120.4
C9A—C8A—S1	123.05 (17)	C15B—C20B—H20B	120.4
C13A—C8A—S1	113.15 (17)	O4—C21—C22	109.66 (19)
C8A—C9A—C10A	120.3 (3)	O4—C21—H211	109.7
C8A—C9A—H9A	119.9	C22—C21—H211	109.7
C10A—C9A—H9A	119.9	O4—C21—H212	109.7
C11A—C10A—C9A	115.3 (3)	C22—C21—H212	109.7
C11A—C10A—H10A	122.4	H211—C21—H212	108.2
C9A—C10A—H10A	122.4	C27—C22—C23	118.9 (2)
C12A—C11A—C10A	125.3 (3)	C27—C22—C21	121.8 (2)
C12A—C11A—H11A	117.4	C23—C22—C21	119.3 (2)
C10A—C11A—H11A	117.4	C24—C23—C22	121.0 (2)
C11A—C12A—C13A	118.6 (3)	C24—C23—H23	119.5
C11A—C12A—H12A	120.7	C22—C23—H23	119.5
C13A—C12A—H12A	120.7	C25—C24—C23	119.6 (2)
C12A—C13A—C8A	117.0 (3)	C25—C24—H24	120.2
C12A—C13A—H13A	121.5	C23—C24—H24	120.2
C8A—C13A—H13A	121.5	C24—C25—C26	120.5 (3)
C9B—C8B—C13B	110.8 (3)	C24—C25—H25	119.8
C9B—C8B—S1	120.9 (2)	C26—C25—H25	119.8
C13B—C8B—S1	127.1 (2)	C25—C26—C27	119.1 (3)
C8B—C9B—C10B	119.3 (3)	C25—C26—H26	120.4
C8B—C9B—H9B	120.3	C27—C26—H26	120.4
C10B—C9B—H9B	120.3	C22—C27—C26	120.9 (2)
C11B—C10B—C9B	128.2 (4)	C22—C27—H27	119.5
C11B—C10B—H10B	115.9	C26—C27—H27	119.5
			
O1—C1—C2—C3	63.0 (2)	C9A—C8A—C13A—C12A	−6.4 (7)
C1—C2—C3—C4	−68.1 (2)	S1—C8A—C13A—C12A	−178.0 (4)
C2—C3—C4—C5	61.6 (2)	C1—S1—C8B—C9B	77.6 (3)
C3—C4—C5—O1	−55.9 (2)	C1—S1—C8B—C13B	−88.4 (4)
C4—C5—O1—C1	61.9 (2)	C13B—C8B—C9B—C10B	−17.1 (6)
C5—O1—C1—C2	−63.1 (2)	S1—C8B—C9B—C10B	174.8 (3)
C5—O1—C1—S1	176.16 (12)	C8B—C9B—C10B—C11B	8.0 (7)
C8A—S1—C1—O1	−80.49 (16)	C9B—C10B—C11B—C12B	6.1 (8)
C8B—S1—C1—O1	−82.40 (16)	C10B—C11B—C12B—C13B	−8.8 (7)
C8A—S1—C1—C2	164.18 (16)	C11B—C12B—C13B—C8B	−2.0 (8)
C8B—S1—C1—C2	162.27 (16)	C9B—C8B—C13B—C12B	15.3 (7)
C7—N1—C2—C3	−30.61 (19)	S1—C8B—C13B—C12B	−177.6 (4)
C14—N1—C2—C3	−178.55 (14)	C7—N1—C14—C15B	−88.23 (17)
C7—N1—C2—C1	−145.7 (2)	C2—N1—C14—C15B	56.42 (17)
C14—N1—C2—C1	66.3 (2)	C7—N1—C14—C15A	−88.61 (17)
O1—C1—C2—N1	173.76 (17)	C2—N1—C14—C15A	56.03 (17)
S1—C1—C2—N1	−68.9 (2)	N1—C14—C15A—C16A	−125.37 (8)
S1—C1—C2—C3	−179.65 (14)	N1—C14—C15A—C20A	46.43 (19)
C7—O2—C3—C4	−154.83 (18)	C20A—C15A—C16A—C17A	3.7 (2)
C7—O2—C3—C2	−31.33 (19)	C14—C15A—C16A—C17A	176.01 (17)
N1—C2—C3—O2	36.67 (17)	C15A—C16A—C17A—C18A	−4.1 (4)
C1—C2—C3—O2	163.95 (15)	C16A—C17A—C18A—C19A	3.4 (5)
N1—C2—C3—C4	164.59 (15)	C17A—C18A—C19A—C20A	−2.5 (5)
O2—C3—C4—O3	−62.1 (2)	C18A—C19A—C20A—C15A	2.4 (5)
C2—C3—C4—O3	178.25 (15)	C16A—C15A—C20A—C19A	−2.9 (4)
O2—C3—C4—C5	−178.73 (16)	C14—C15A—C20A—C19A	−174.7 (2)
C1—O1—C5—C6	−175.85 (16)	N1—C14—C15B—C16B	−150.3 (2)
O3—C4—C5—O1	−176.08 (15)	N1—C14—C15B—C20B	41.6 (2)
O3—C4—C5—C6	64.4 (2)	C20B—C15B—C16B—C17B	−5.1 (5)
C3—C4—C5—C6	−175.48 (17)	C14—C15B—C16B—C17B	−173.2 (3)
C21—O4—C6—C5	75.1 (2)	C15B—C16B—C17B—C18B	3.8 (6)
O1—C5—C6—O4	51.8 (2)	C16B—C17B—C18B—C19B	−3.1 (7)
C4—C5—C6—O4	173.44 (16)	C17B—C18B—C19B—C20B	3.7 (7)
C14—N1—C7—O5	−16.3 (3)	C18B—C19B—C20B—C15B	−4.8 (6)
C2—N1—C7—O5	−166.1 (2)	C16B—C15B—C20B—C19B	5.5 (5)
C14—N1—C7—O2	162.90 (14)	C14—C15B—C20B—C19B	173.5 (3)
C2—N1—C7—O2	13.1 (2)	C6—O4—C21—C22	−172.10 (18)
C3—O2—C7—O5	−168.81 (19)	O4—C21—C22—C27	−1.1 (3)
C3—O2—C7—N1	12.0 (2)	O4—C21—C22—C23	178.6 (2)
C1—S1—C8A—C9A	51.4 (3)	C27—C22—C23—C24	0.0 (4)
C1—S1—C8A—C13A	−136.9 (3)	C21—C22—C23—C24	−179.7 (2)
C13A—C8A—C9A—C10A	7.4 (6)	C22—C23—C24—C25	−1.4 (4)
S1—C8A—C9A—C10A	178.2 (3)	C23—C24—C25—C26	1.4 (5)
C8A—C9A—C10A—C11A	−4.0 (6)	C24—C25—C26—C27	0.0 (5)
C9A—C10A—C11A—C12A	0.1 (7)	C23—C22—C27—C26	1.5 (4)
C10A—C11A—C12A—C13A	0.8 (8)	C21—C22—C27—C26	−178.8 (3)
C11A—C12A—C13A—C8A	2.2 (8)	C25—C26—C27—C22	−1.5 (5)

### Hydrogen-bond geometry (Å, °)

**Table:**

*D*—H···*A*	*D*—H	H···*A*	*D*···*A*	*D*—H···*A*
O3—H3O···O5^i^	0.84	1.98	2.774 (2)	158.

Symmetry codes: (i) −*x*, *y*+1/2, −*z*+1.

## References

[bb1] Benakli, K., Zha, C. & Kerns, R. J. (2001). *J. Am. Chem. Soc.* **123**, 9461–9462. 10.1021/ja0162109 11562237

[bb2] Blessing, R. H. (1995). *Acta Cryst.* A**51**, 33–38. 10.1107/s0108767394005726 7702794

[bb3] Boysen, M., Gemma, E., Lahmann, M. & Oscarson, S. (2005). *Chem. Commun.* pp. 3044–3046. 10.1039/b503651h 15959579

[bb4] Burla, M. C., Caliandro, R., Camalli, M., Carrozzini, B., Cascarano, G. L., De Caro, L., Giacovazzo, C., Polidori, G. & Spagna, R. (2005). *J. Appl. Cryst.* **38**, 381–388.

[bb5] Cremer, D. & Pople, A. (1975). *J. Am. Chem. Soc.* **97**, 1354–1358.

[bb6] Crich, D. & Vinod, A. U. (2005). *J. Org. Chem.* **70**, 1291–1296. 10.1021/jo0482559 15704963

[bb7] Farrugia, L. J. (1997). *J. Appl. Cryst.* **30**, 565.

[bb14] Flack, H. D. (1983). *Acta Cryst.* A**39**, 876–881.

[bb8] Geng, Y., Zhang, L.-H. & Ye, X.-S. (2008). *Tetrahedron*, **64**, 4949–4958.

[bb9] Manabe, S., Ishii, K. & Ito, Y. (2006). *J. Am. Chem. Soc.* **128**, 10666–10667. 10.1021/ja062531e 16910646

[bb10] Otwinowski, Z. & Minor, W. (1997). *Methods in Enzymology*, Vol. 276, *Macromolecular Crystallography*, Part A, edited by C. W. Carter Jr & R. M. Sweet, pp. 307–326. New York: Academic Press.

[bb11] Rigaku/MSC (2005). *CrystalClear SM.* Rigaku/MSC Inc., The Woodlands, Texas, USA.

[bb12] Satoh, H., Hutter, J., Luthi, H.-P., Manabe, S., Ishii, K. & Ito, Y. (2008). In preparation.

[bb13] Sheldrick, G. M. (2008). *Acta Cryst.* A**64**, 112–122. 10.1107/S0108767307043930 18156677

